# Family Clustering of Viliuisk Encephalomyelitis in Traditional and New Geographic Regions

**DOI:** 10.3201/eid1309.061585

**Published:** 2007-09

**Authors:** Vsevolod A. Vladimirtsev, Raisa S. Nikitina, Neil Renwick, Anastasia A. Ivanova, Al’bina P. Danilova, Fyodor A. Platonov, Vadim G. Krivoshapkin, Catriona A. McLean, Colin L. Masters, D. Carleton Gajdusek, Lev G. Goldfarb

**Affiliations:** * Institute of Health–Sakha (Yakut) Republic, Yakutsk, Russian Federation; † Columbia University Medical Center, New York, New York, USA; ‡ University of Melbourne, Parkville, Victoria, Australia; § Institut Alfred Fessard, Gif-sur-Yvette, France; ¶ National Institutes of Health, Bethesda, Maryland, USA

**Keywords:** Viliuisk encephalomyelitis, Yakut (Sakha) people, Siberia, Viliui River, Familial aggregation, research

## Abstract

Viliuisk encephalomyelitis is an acute, often fatal, meningoencephalitis that tends to develop into a prolonged chronically progressive panencephalitis. Clinical, neuropathologic, and epidemiologic data argue for an infectious cause, although multiple attempts at pathogen isolation have been unsuccessful. To assess mechanisms of disease transmission and spread, we studied 6 multiplex families. Secondary cases occurred among genetically related and unrelated persons in a setting of prolonged intrahousehold contact with a patient manifesting the disease. Transmission to unrelated persons was documented in a densely populated region around the city of Yakutsk in which Viliuisk encephalomyelitis had not been previously known. Initially identified in a small Yakut-Evenk population on the Viliui River of eastern Siberia, the disease subsequently spread through human contacts to new geographic areas, thus characterizing Viliuisk encephalomyelitis as an emerging infectious disease.

## Abstract 2

Transmission occurs through patient contact; human migration from disease-endemic villages leads to disease emergence in new communities.

Viliuisk encephalomyelitis (VE) is clinically and pathologically defined as an acute meningoencephalitis that progresses to a more prolonged panencephalitic syndrome (*1*–*3*). In a small number of patients, the initial acute phase had gone undetected. Cerebrospinal fluid (CSF) pleocytosis is present during the acute phase and subsequent progressive stages of illness. Postmortem examination identifies diffuse infiltration of the meninges with mononuclear and plasma cells; multiple micronecrotic lesions in the brain parenchyma are surrounded by T and B lymphocytes and reactive astrocytes (*4*,*5*). These disease characteristics suggest that VE is an infectious disease, although the causative agent has not been identified and the mechanisms of disease transmission and spread remain unknown.

Strong evidence exists that VE is disseminating through migration of affected persons from villages along the Viliui River in Eastern Siberia, where VE has been endemic for at least a century, to densely populated regions around the city of Yakutsk (*6*), located ≈4° (450 km) below the ArcticCircle (*7*,*8*). This characterizes VE as an emerging infectious disease. From 1950 to 1959, all known VE patients were born in villages in the Viliui valley (*1*), whereas in 1970–1979, 32% of VE patients were identified in previously unaffected regions of Lena and Aldan valleys in the vicinity of Yakutsk (*7*). VE prevalence in these newly affected regions remained stable at 35% in the decade1980–1989 and has been slowly declining since the mid-1990s (*9*).

A preliminary study of familial aggregation of VE patients was based on data collected in the 1950s, 1960s, and 1970s (*7*,*8*). Of 194 VE-affected families, 27 had multiple VE cases, 2 per family in 24 families and 3 per family in 3. Secondary cases occurred in 11 full siblings, 3 children of the index case-patient, a niece, half-sibling, a cousin, 3 spouses, and 10 adopted children or other genetically unrelated persons living in the same household (*7*). The phenomenon of VE clustering in the affected households was verified by statistical analysis. Statistical analyses indicated that that clustering of >2 cases per family occurred more frequently than should be expected on the basis of family size and VE prevalence rates (χ^2^ test, p = 0.00036) (*7*,*8*).

We sought to document and better characterize the phenomenon of VE clustering in the affected households. We conducted a detailed study of 5 families living in villages along the Viliui River and of 1 family in the region surrounding the city of Yakutsk, to which the disease has recently spread.

## Patients and Methods

VE patients were identified by physicians from neurology services established in the 1950s in Viliuisk (*1*,*3*) and Yakutsk (*6*). Early detection and frequent follow-up of VE patients were accomplished by village-to-village searches and periodic hospitalizations. VE diagnoses were made according to established clinical (*2*,*3*) and pathologic (*4*) criteria; standardized neurologic assessment was performed on all patients, and neuropathologic examination was performed by 3 independent groups (*4*,*5*,*10*). In the 50-year period between 1950 and 2000, 301 patients were identified. Families with 2 or more patients were repeatedly studied in the villages by visiting epidemiologist/neurologist teams, which collected epidemiologic data and reviewed medical histories. Six well-characterized VE-affected families with 2 or 3 patients were included in the current study; neuropathologic examination was performed on 1 patient from each family.

Studies were conducted under clinical protocols approved by the Institutional Review Boards of the Institute of Health, Sakha (Yakut) Republic, and the US National Institutes of Health. The protocol was subsequently reviewed and approved by the Office of Protection from Research Risks, US Department of Health and Human Services (OPRR-S-16078–01). Informed consent was obtained for each element of this study.

## Results

Five families originated from settlements in a high-incidence middle Viliui region with a rural population of 10,000, composed of remnants of the indigenous Tungus (Evenk) tribes that have been largely assimilated by the dominant Yakut (Sakha) people. Families 1, 3, and 4 were identified in villages around Lake Mastakh, the peak VE-endemic area; family 2 was from a village near the town of Viliuisk; family 5 was identified in a settlement 100 km down the Viliui River; and family 6 was studied near the capital city of Yakutsk, ≈400 km southeast of Lake Mastakh (Figure). The population in this central region is ethnic Yakut (Sakha).

**Figure:**
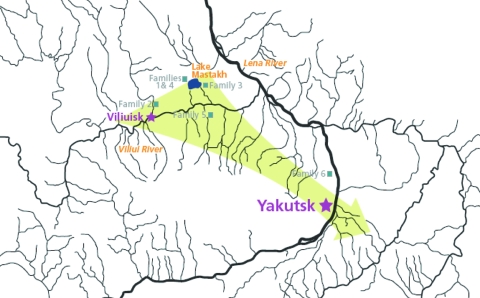
Location of families with Viliuisk encephalomyelitis characterized in this report. Arrow indicates the general direction of Viliuisk encephalomyelitis dispersion from traditional disease-endemic areas on the Viliui River to densely populated regions of the Sakha (Yakut) Republic around the capital city of Yakutsk.

### Family 1

Family 1 consisted of 2 parents and 5 siblings (3 daughters and 2 sons).VE developed in each daughter; no other VE patients were identified in the extended pedigree of this family. In the index case-patient (patient 1-1, Table 1), the second-born daughter, high fever, severe headache, myalgia, chills, and double vision developed when she was 16 years of age in 1954. She was admitted to the hospital in a coma and remained drowsy and mentally and physically slow for 6 weeks. Six months later, she had a relapse of a febrile illness with persistent neck stiffness, Kernig sign, and repetitive generalized seizures. After the acute phenomena subsided, she showed evident cognitive impairment, upper motor neuron pattern of weakness in limb muscles, hyperactive deep tendon reflexes, bilateral Babinski sign, dysarthric speech, and spastic gait. Ten months after disease onset, she exhibited dementia dysarthria and spastic quadriparesis. The disease progressed relentlessly, and the patient died 18 months after the onset of symptoms. Cell count and protein concentration level were consistently abnormal during the entire course of illness (the results of CSF testing and neuropathologic findings are listed in Table 2).

**Table 1:** Clinical features of Viliuisk encephalomyelitis in affected members of 6 families*

Feature	Family 1			Family 2		Family 3		Family 4			Family 5		Family 6		
	1-1	1-2	1-3	2-1	2-2	3-1	3-2	4-1	4-2	4-3	5-1	5-2	6-1	6-2	6-3
Sex	F	F	F	F	F	F	M	M	F	F	F	F	M	F	F
Age at onset, y	16	38	26	16	44	31	21	51	47	36	20	34	46	37	53
Febrile stage of illness															
Duration, wk	7	11	2	2	10	ND	2	ND	ND	4	ND	1	ND	6	26
Maximum temperature, ^o^C	40.5	39	38	37.9	39.8		39.0			39.0		ND		38.5	38.8
Reduced level of consciousness	++	+++		+	+++		+			++				+	+++
Generalized seizures	++			+						+					
Neck rigidity and Kernig sign	+	++		+	++		+			+					+
Nausea, vomiting	+	++	+		++									++	+
Upper motor neuron pattern of muscle weakness	+				+					+				+	
Relapse of febrile illness	+	+								+					+
Outcome	a	d	a	a	d	a	a	a	a	a	a	a	a	a	d
Advanced disease															
Cognitive decline	++		+	++		m.i.	++	++	++	++	++	++	+	++	
Apathy	+			+			++		+	+		+		+	
Dysarthria	+++		++	++		++	+	++	+	++			+	+	
Dysphagia							+	+		+		+			
Brisk deep tendon reflexes	++		++	++		++	++	++	+	++	+	++	+	++	
Spastic tetraparaparesis	+++		+	++		+	++	+	++	+++	–	+	+	–	
Increased muscle tone	+		+	+		+	++	+	+	++	–	+		+	
Babinski sign	+		+	+			+	+	+	+		+			
Spastic gait	+++		++	++		+	+++	+	+	+	+	+	+	+	
Falls	+			++			+			+			+		
Sphincter dysfunction	+			++			++					+			
Extrapyramidal rigidity	+		+	++		+	++	++	+	+			+	+	
Obesity	+		+	+										+	
Cachexia		+			+					++					+
Overall duration of illness	18 mo	11 wk	A	48 mo	10 wk	17 y	24 mo	9 y	6 y	11 mo	11 y	23 mo	13 y	27 mo	26 wk

*ND, not documented; m.i., memory impairment; +, mild; ++, moderate; +++, strong; –, not expressed; d, died within acute phase; a, advanced disease developed; A, alive.

**Table 2:** Laboratory investigations and postmortem findings in Viliuisk encephalomyelitis patients from 6 families*

Laboratory and postmortem findings	Family 1			Family 2		Family 3		Family 4			Family 5		Family 6		
	1-1	1-2	1-3	2-1	2-2	3-1	3-2	4-1	4-2	4-3	5-1	5-2	6-1	6-2	6-3
Cerebrospinal fluid															
Cell count (cells/μL)	20–102	8	3	45–62	15–44	2	11–58	25	17	4– 18	NA	12	NA	17–65	11–27
Predominant cell type	Lym			Lym	Lym		Lym							Lym	Lym
Protein (mg/dL)	150	166	23	480	99	16	99	33	66	132		66		66	99
Bacterial culture	Neg	Neg	NA	Neg	Neg	NA	Neg	NA	NA	NA		NA		Neg	Neg
Postmortem															
Inflamed meninges		+			+		+		+			+			+
Micronecrotic lesions in the brain parenchyma		+			+		+		+			+			+

*NA, data not available; Lym, lymphocytes; Neg, negative; +, observed.

The eldest sister (patient 1-2) became acutely ill at 38 years of age, 15 years after the death of patient 1-1. She exhibited high fever, chills, headache, myalgia, nausea, and frequent vomiting and was comatose when admitted to the hospital; examination findings included dysphagia, neck stiffness, and Kernig sign. The patient’s condition rapidly deteriorated, and she died on day 75 of illness. On postmortem examination, the meninges were thickened and infiltrated with mononuclear, plasma, and polymorphonuclear cells. Inflammatory changes were especially pronounced in the meninges overlying the affected cortical areas. In the brain, multiple widespread micronecrotic foci surrounded by inflammatory infiltrates were observed throughout the cerebral cortex, basal ganglia, cerebellum, and brain stem. The infiltrates were composed of lymphocytes (predominantly T-cells with occasional B-cells), microglial cells, macrophages, and reactive astrocytes. Perivascular cuffs consisting of mononuclear cells were widespread in the affected areas. Diffuse neuronal loss was observed within and outside the affected areas. Mild fibrillary gliosis in its early stages was present in the affected areas of pontine nuclei and inferior olives (*5*).

The youngest sister (patient 1-3) had a 2-week febrile illness with headache, chills, and insomnia at the age of 22, 11 years after the death of patient 1-1. Subsequently, cognitive decline, muscle stiffness, dysarthria, and spastic gait developed. Disease progression slowed 10 years after the onset, and she is still alive in her 39th year of illness.

### Family 2

In family 2, which consisted of parents and 6 siblings (3 sisters and 3 brothers), an acute febrile illness developed in the second-born sister (patient 2-1) when she was 16 years of age. She recovered, but 3 months after the onset became slow mentally and clumsy physically, with spastic gait and frequent falls. Seven months after the disease onset, she was admitted to the hospital with recurrent generalized tonic-clonic seizures, cognitive decline, dysarthria, brisk tendon reflexes throughout, flexor and extensor pathologic reflexes, increased muscle tone, and spastic gait. The disease relentlessly progressed; 28 months after the disease onset, she was obese, demented, dysarthric, with muscle weakness and spastic gait. She died 48 months after symptom onset. CSF tests suggested inflammatory brain disease (Table 2).

In the oldest sister (patient 2-2) a sudden febrile illness developed when she was 44 years of age, 16 years after her younger sister’s death. She sought treatment for severe headache, confusion, nausea, and vomiting and had meningeal signs on examination. During hospitalization, she remained febrile, and hyperactive tendon reflexes developed along with ankle clonus, bilateral Babinski sign, progressive dysphagia, and respiratory failure. The patient died 72 days after disease onset. Results of postmortem examination were consistent with the diagnosis of acute meningoencephalitic form of VE (Table 2). No other VE patients are known among the immediate or distant family members.

### Family 3

Family 3 comprised an affected mother (patient 3-1) and her 3 sons; VE developed in the mother in 1953 and in her oldest son (patient 3-2) in 1973. Patient 3-1 had an insidious onset of muscle stiffness, gait disturbance, dysarthria, and memory impairment at the age of 31. On examination, she had severe dysarthria, moderate muscle weakness in the lower limbs, brisk tendon reflexes, flexor pathologic signs, spastic gait, and bilateral mild muscle atrophy of the hand and forearm muscles. The disease progressed slowly, and the patient died 17 years after onset.

In patient 3-2, a 2-week febrile illness developed when he was 21 years of age, with severe headache and dizziness, 2 years after his mother’s death. He recovered but was unable to do any work on his family’s farm. The following year, rapidly progressive dementia, dysarthria, muscle stiffness, and spasticity developed. On examination, he had brisk tendon reflexes with ankle clonus, spastic quadriparesis, and decreased proprioceptive sensation in the lower limbs with preserved vibration sense. Later he had bulbar symptoms and atrophy of small muscles of the hands and feet and died in respiratory distress 24 months after disease onset. A cranial computed tomographic scan showed cortical atrophy and diffuse leptomeningeal enhancement over the parietal and occipital lobes.

Study of the neuropathologic features of patient 3-2 showed thickened and cloudy meninges and microscopically diffuse infiltration with mononuclear, plasma, and polymorphonuclear cells. Organized necrotic foci with central lysis of tissue and adjacent reactive gliosis were abundant in the brain parenchyma. Small vessels within and adjacent to these foci showed endothelial cell proliferation and perivascular cuffs of T-lymphocytes. Some areas showed confluence of many recent and old necrotic lesions, leading to an extensive destruction in all cortical laminae, reactive fibrillary gliosis, and secondary demyelination in the underlying white matter. Organizing and active inflammatory foci were present in the putamen, the globus pallidus, and claustrum. Examination of the spinal cord showed gross degeneration of corticospinal tracts and less pronounced degeneration in Goll columns. Perivascular cuffs of mononuclear cells were frequently found in the degenerating tracts and the adjacent areas (*5*,*11*).

### Family 4

Family 4 comprised a woman with 3 sons and a daughter from consecutive marriages; VE developed in the eldest son (patient 4-1), his half-sibling (patient 4-2), and 1 of his 4 children (patient 4-3). Patient 4-1 had a short febrile episode in 1952 at the age of 51 years; the following year, muscle stiffness and speech and gait abnormalities developed. Two years later, he had overt dementia, characteristic dysarthria, and spastic gait. He died 9 years after the onset of neurologic symptoms. The CSF specimen analyzed during the second year of illness showed pleocytosis (Table 2).

Patient 4-2 became visibly affected in 1965, shortly before the age of 47, 4 years after her half-brother’s death. The illness had an insidious onset, beginning with clumsiness and decreasing muscle strength in the lower limbs. Further progression led to intellectual decline and slurred dysarthric speech. On examination, she was mute and moved slowly due to spasticity predominantly in the lower limbs. She died 6 years after the disease onset. Postmortem examination showed characteristic necrotic lesions with marked central lysis of tissue, reactive gliosis, and secondary demyelination in the underlying white matter. T-lymphocytes and rod-shaped microglia were present within the gray and white matter (*5*).

Sudden acute disease developed in patient 4-3 in 1971 when she was 36 years of age, 10 years after her father’s death. At hospital admission, she was unresponsive, febrile, and had neck stiffness and Kernig sign. She remained confused and lethargic for 4 weeks and thereafter showed significant memory loss and signs of spasticity. Nine weeks later, her condition worsened; she remained unconscious on life support and died 11 months after disease onset.

### Family 5

Family 5 consisted of parents and 5 siblings (3 sisters and 2 brothers); VE developed in 2 sisters. The younger of the 2 affected sisters (patient 5-1) was the first to exhibit a gradual cognitive decline, spastic gait, dysphonia, and slurred speech, but not overt dysarthria, at the age of 20 years in 1957. The disease progressed very slowly; she became globally demented and died 11 years after the onset.

Her older sister (patient 5-2) had a short flulike illness at the age of 34 years, while the index patient was still alive. This was followed by cognitive decline and slowness of movements. Examination during the second year of illness showed deepening dementia, moderate muscle weakness in the upper and lower limbs, increasing spasticity, and urine incontinence. The disease rapidly progressed, and the patient died 23 months after the onset of flulike symptoms. Postmortem study showed marked brain atrophy and widened ventricles and multiple fresh and organized necrotic foci surrounded by spongiform degeneration and reactive gliosis. Perivascular cuffs consisting of lymphocytes, plasma, and polymorphonuclear cells were widespread in the affected portions of the cerebral cortex, subcortical ganglia, cerebellum, and inferior olives (*11*). No other members of the extended family were known to be affected.

### Family 6

Family 6 included a man and his 2 consecutive wives; VE developed in all. The man (patient 6-1) was born in a disease-endemic region on the Viliui River near the village where family 5 was identified. At the age of 25 years, he moved to a small settlement in the suburbs of the capital city of Yakutsk (Figure). This migrant worker lived for many years with a local Yakut family and married a young member of the adopting family (patient 6-2). They had a healthy child. Around the time of symptom onset in 1959, patient 6-1 moved to another village in this same area where his second wife, a local woman (patient 6-3) took care of him during his illness. At this time (late 1950s), no VE cases were known in this part of the country; neither the local people nor the practicing physicians in the region had ever seen or heard of VE.

The illness in patient 6-1 had an insidious onset when the patient was 46 years of age; he exhibited increasing clumsiness and loss of muscle strength in the lower limbs. A year later, his speech became dysarthric and his gait slow and spastic. He lost the ability to walk and speak around the 10th year of illness and died 13 years after the disease onset. The patient was repeatedly studied at the regional hospital.

In patient 6-2, an acute disease developed when she was 37 years of age, 17 years after her relationship with patient 6-1 ended. She sought treatment for fever, headache, dizziness, chills, nausea and frequent vomiting, diplopia, and abnormal behavior. Ten weeks later, she showed a substantial intellectual decline, slowness of movements, spasticity, limb ataxia, and slow abnormal gait with frequent falls. Six months after the disease onset, she gained weight, became globally demented, and exhibited dysarthria, muscle weakness in the upper and lower limbs, and spastic gait. She died 27 months after the onset of symptoms. CSF studies during the illness showed inflammatory response (Table 2).

Patient 6-3 became acutely ill at the age of 53 years, 2 years after the death of patient 6-1. She experienced severe headache, chills, and nausea and vomiting. Six weeks after symptom onset, she had a second episode of febrile illness and was admitted to the hospital with high temperature; she was also disoriented and aggressive. Her condition worsened and she died 26 weeks after disease onset. On postmortem examination, the meninges were infiltrated; the cerebral cortex and other gray matter structures contained widespread micronecrotic foci surrounded by inflammatory infiltrates with a tendency for these lesions to be replaced with gliofibrotic scars (*5* [Case 1], *10*).

## Discussion

We studied 6 families that included 15 patients with a definitive diagnosis of VE, according to published clinical and neuropathologic criteria (*3*,*4*). The abrupt febrile disease onset in most of the studied patients, the developing meningoencephalitis with CSF pleocytosis, and inflammatory changes systematically found in the brain tissue strongly suggest that VE is an infectious disease. Furthermore, VE patients show evidence for intrathecal immunoglobulin G synthesis, which correlates with the clinical manifestations (*12*). The prolonged occurrence of increased cell count and elevated protein concentrations in the CSF, up to 5–6 years from the disease onset, and the development of chronically progressive dementia and movement abnormalities, suggest that the pathogen is an unconventional organism, which may explain the failure of its isolation and identification (*7*).

Although the occurrence of VE exclusively in the Yakut (Sakha) population may suggest a genetically determined susceptibility of the indigenous Viliui population, segregation analysis excluded Mendelian inheritance (*7*,*8*). A recent case-control study discovered allelic associations (p<0.05) between interferon-γ (IFN-γ) gene polymorphisms and VE susceptibility. Notably, allelic association was found only in older patients who survived the acute disease phase, which suggests that IFN-γ variants may be predisposing to the development of chronic VE (T. Oleksyk, pers. comm.). The spread of VE to new geographic regions argues against the view that some Siberian subpopulations are more susceptible to VE than others.

Five families with >1 VE patient were identified and studied in a high-incidence mid-Viliui region, but the most interesting data were obtained from studies of family 6 in a region located 400 km away from the peak VE-endemic region around Lake Mastakh, where VE has not been previously known. Transmission to unrelated persons in a new environment confirms that prolonged intrahousehold contact is a significant risk factor. VE transmission to unrelated persons was observed in several other families, but the clinical, pathologic, or epidemiologic documentation is insufficient.

Our analysis shows that the most severe disease resulting in death after an acute illness occurred in secondary but not primary cases (families 1, 2, 4, and 6). This discordance between the primary and secondary cases within a family suggests that transmission of infection in the setting of close intrahousehold contact may result in shorter incubation times and faster progression of the illness. Variability of VE phenotypic manifestations has been reported (*7*), but it was instructive to observe extremely diverse outcomes in members of the same family.

In summary, VE is a unique meningoencephalitis occurring in the Yakut (Sakha) population of Eastern Siberia. Although the pathogen has not been identified, clinical and pathologic phenomena described here indicate that the only plausible explanation is underlying infection. Analysis of case clustering in 6 families supports the view that VE can be transmitted in a setting of a prolonged intrahousehold contact with a patient manifesting the disease. To our knowledge, this is the first report of VE transmission to unrelated persons, occurring in a region in which VE has not been previously known. The spread from high-incidence foci along the Viliui Valley to new geographic areas strongly indicates that VE is an emerging infectious disease.

## References

[1] Petrov PA. Viliuisk encephalitis (encephalomyelitis) [in Russian]. S.S. Korsakov’s Journal of Neurology and Psychiatry. 1958;58:669–74.

[2] Shapoval AN. Viliuisk encephalomyelitis. Yakutsk (Russia): Yakutsk Publishing House;1959.

[3] Petrov PA. Viliuisk encephalitis (encephalomyelitis). Yakutsk (Russia): Yakutsk Publishing House; 1964.

[4] Savinov AP, Zubri GL, Robinzon IA, Iurovetskaya AL. Pathomorphology of the central nervous system in Viliuisk encephalomyelitis. In: Current issues of virology and prevention of viral encephalitides. Moscow: Academy of Medical Sciences; 1972. Vol. 17. p. 203–5.

[5] McLean CA, Masters CL, Vladimirtsev VA, Prokhorova IA, Goldfarb LG, Asher DM, Viliuisk encephalomyelitis—review of the spectrum of pathological changes. Neuropathol Appl Neurobiol. 1997;23:212–7. 10.1111/j.1365-2990.1997.tb01204.x 9223130

[6] Vladimirtsev AI. Chronic Yakut (Viliuisk) encephalomyelitis during 12 years in records of the Neurology Service of the Republican Hospital. Bulletin of the Yakut Republican Hospital, Yakutsk. 1964;9:97–106.

[7] Goldfarb LG, Gajdusek DC. Viliuisk encephalomyelitis in the Iakut population of Siberia. Brain. 1992;115:961–78. 10.1093/brain/115.4.961 1393513

[8] Goldfarb LG, Fedorova NI, Chumakov MP, Petrov PA, Vladimirtsev AI, Ivanova AI. Relationship of hereditary and environmental factors in the etiology of Viliuisk encephalomyelitis. 1. Affected families [in Russian]. Genetika. 1979;15:1502–12. 478288

[9] Alekseev VP, Krivoshapkin VG, Makarov VN. Geography of Viliuisk encephalomyelitis. Yakutsk (Russia): Institute of Health, Yakutsk; 2000. p. 1–72.

[10] Avtsyn AP, Prokhorova IA, Zhavoronkov AA, Goldfarb LG. Clinical characterization and histopathology of Viliuisk encephalomyelitis [in Russian]. S.S. Korsakov’s Journal of Neurology and Psychiatry. 1983;83:204–8. 6858478

[11] Catalogue of cases of Viliuisk encephalomyelitis studied in Iakutia, 1967–1975. Moscow: Academy of Medical Science; 1976.

[12] Green AJE, Sivtseva TM, Danilova AP, Osakovsky VL, Vladimirtsev VA, Zeidler M, Viliuisk encephalomyelitis: intrathecal synthesis of oligoclonal IgG. J Neurol Sci. 2003;212:69–73. 10.1016/S0022-510X(03)00107-2 12810001

